# Key issues in Japan’s public health centers to prepare for future pandemics: a text mining study using a topic model

**DOI:** 10.1186/s12913-024-11094-w

**Published:** 2024-05-17

**Authors:** Kosuke Sakai, Yu Igarashi, Shuji Tounai, Chika Shirai, Yoko Tsurugi, Fumihiko Kakuno, Yukako Komasa, Maya Fujimura, Mika Uruha, Koji Mori, Seiichiro Tateishi

**Affiliations:** 1 Department of Occupational Health Practice and Management, Institute of Industrial Ecological Sciences, University of Occupational and Environmental Health, Japan, 1-1 Iseigaoka Yahatanishi-Ku, Kitakyushu-Shi, Fukuoka-Ken 807-8555 Japan; 2 Disaster Occupational Health Center, Institute of Industrial Ecological Sciences, University of Occupational and Environmental Health, Japan, 1-1 Iseigaoka Yahatanishi-Ku, Kitakyushu-Shi, Fukuoka-Ken 807-8555 Japan; 3 Oita Prefecture Welfare and Public Health Department, Otemachi, Oita-shi, Oita-ken 870-0022 Japan; 4 Hirakata City Public Health Center, 2-2-2 Ogaitocho, Hirakata-shi, Osaka-fu 573-0027 Japan; 5 Kikuchi Public Health Center, Kumamoto, 1272-10 Waifu, Kikuchi-shi, Kumamoto-ken 861-1331 Japan; 6 Shiga prefecture Department of Public Health and Medical Welfare, 4-1-1 Kyomachi, Otsu-Shi, Shiga-Ken 520-8577 Japan; 7 Department of Community and Global Health, Graduate School of Medicine, The University of Tokyo, 7-3-1 Hongo, Bunkyo-ku, Tokyo-to, 113-0033 Japan; 8 School of Medicine, University of Occupational and Environmental Health, Japan, 1-1 Iseigaoka Yahatanishi-ku, Kitakyushu-shi, Fukuoka-ken 807-8555 Japan

**Keywords:** COVID-19, Public health systems research, Pandemic, Data mining

## Abstract

**Background:**

In Japan, over 450 public health centers played a central role in the operation of the local public health system in response to the COVID-19 pandemic. This study aimed to identify key issues for improving the system for public health centers for future pandemics.

**Methods:**

We conducted a cross-sectional study using an online questionnaire. The respondents were first line workers in public health centers or local governments during the pandemic. We solicited open-ended responses concerning improvements needed for future pandemics. Issues were identified from these descriptions using morphological analysis and a topic model with KHcoder3.0. The number of topics was estimated using Perplexity as a measure, and Latent Dirichlet Allocation for meaning identification.

**Results:**

We received open-ended responses from 784 (48.6%) of the 1,612 survey respondents, which included 111 physicians, 330 nurses, and 172 administrative staff. Morphological analysis processed these descriptions into 36,632 words. The topic model summarized them into eight issues: 1) establishment of a crisis management system, 2) division of functions among public health centers, prefectures, and medical institutions, 3) clear role distribution in public health center staff, 4) training of specialists, 5) information sharing system (information about infectious diseases and government policies), 6) response to excessive workload (support from other local governments, cooperation within public health centers, and outsourcing), 7) streamlining operations, and 8) balance with regular duties.

**Conclusions:**

This study identified key issues that need to be addressed to prepare Japan’s public health centers for future pandemics. These findings are vital for discussions aimed at strengthening the public health system based on experiences from the COVID-19 pandemic.

**Supplementary Information:**

The online version contains supplementary material available at 10.1186/s12913-024-11094-w.

## Background

The novel coronavirus disease 2019 (COVID-19) emerged as a threat to public health [1]. The development and widespread adoption of treatment methods and vaccines have since reduced the mortality rate, and by May 2023, the World Health Organization declared an end to the public health emergency of international concern, indicating a shift towards crisis resolution [2, 3]. Japan likewise downgraded its disease handling protocol for COVID-19 from Class 2, including tuberculosis and SARS, to Class 5, including seasonal influenza, under the Act on the Prevention of Infectious Diseases and Medical Care for Patients with Infectious Diseases [4]. In the preceding three years, nations globally fortified the functionality of their public health institutions in a bid to suppress the pandemic [5].

In Japan, public health centers, under the jurisdiction of the local governments, played a pivotal role in mitigating the pandemic’s impacts [6, 7]. Even prior to the pandemic, these health centers were instrumental in sustaining local public health [8]. Based on the Community Health Act of 1994, Japan had 469 public health centers in prefectures, ordinance-designated cities, and core cities in 2020 [9]. Responding to infectious disease outbreaks was one of their many responsibilities for public health. The public health centers provided services such as infectious disease monitoring, HIV/AIDS and intractable disease management, mental health services, elderly health care, maternal and child health care, as well as food hygiene, sanitation, and medical care supervision [8, 10]. Additionally, these centers served as key response hubs during health crises, including natural disasters and infectious disease outbreaks [11, 12]. When infectious diseases, such as measles, rubella, and tuberculosis, emerged, public health centers swiftly responded [10]. Therefore, from the early stages of the COVID-19 outbreak, these centers conducted epidemiological surveys, contact tracing, consultations for contacts and returnees from abroad, and managed the expansion and operation of PCR testing [13–16]. As the infection spread domestically, their responsibilities expanded to include monitoring the health of home-care patients and coordinating their treatment in hotels or medical institutions.

The public health centers played a central role in the operation of local public health during this pandemic [17]. As the pandemic’s peaks arrived intermittently, the staff, particularly public health nurses, faced increasing burdens, leading to instances of depression and burnout [18, 19]. It has been reported that both a shortage of available human resources and inadequate support during the pandemic contributed to their exhaustion [19]. The nearly three-year experience served as a lesson, prompting a reconsideration of the existing public health system in preparation for future pandemics.

To prepare for a potential resurgence of new infectious diseases, it is crucial to utilize the lessons learned from this pandemic to strengthen future responses. Several reports have attempted to review the current pandemic from governmental and expert perspectives [9, 11]. However, to our knowledge, there are no articles summarizing the issues that need to be addressed in future public health center systems from the viewpoint of those who have been directly responding to the pandemic at public health centers. In addition to expert reviews, it would be beneficial to collect a wide range of opinions from practitioners involved in the response and summarize discussion topics for the future public health center system. Hence, this study aims to identify and organize the issues that should be considered in preparation for future pandemic crises from practitioners’ perspectives.

## Methods

### Study design

We carried out a cross-sectional study utilizing a questionnaire directed at workers in local governments and public health centers throughout Japan. The respondents were asked to provide unguided descriptions of the improvements needed in the future health center system, based on their response to the epidemic. To structure the issues for future discussion, we analyzed the descriptions using morphological analysis and a topic model in this study.

### Selection of respondents in analysis

We recruited respondents via the managers of local governments and public health centers. We distributed letters of invitation to partake in the study to members of the Japanese Association of Health Directors of Prefectural Governments and the Japanese Association of Public Health Center Directors. We invited all managers of 468 public health centers, 47 local governments, and 20 government-designated cities, which covers all institutions in Japan, to participate in the study. The managers disseminated the invitation within their institutions, requesting workers to respond to the online questionnaire. The eligibility criterion for respondents was involvement in the COVID-19 pandemic response for at least six months as their primary duty. To collect a wide range of perspectives, managers were asked to encourage responses from individuals of various ages and professions, including physicians, veterinarians, public health nurses, medical nurses, other professionals, and administrative staff. We targeted approximately 10 respondents per institution to minimize the burden of response and to prevent response bias towards a few institutions. Respondents completed the questionnaire by scanning a QR code or URL, which redirected them to a Microsoft Form. The response period spanned from December 2022 to January 2023.

### Data collection

The online questionnaire consisted of questions regarding the characteristics of the respondents and public health center systems that should be discussed in preparation for the future. The questions related to the characteristics of the respondents were: sex (man, woman), age (20 s, 30 s, 40 s, 50 s, 60 +), length of continuous employment (0–5 years, 6–10 years, 11–15 years, 16 + years), occupation (public health nurse or midwife, doctor or dentist, other technicians, administrative staff), job position (manager, non-manager), workplace (public health center, local government, other) areas of jurisdiction (47 prefectures). To collect text data in line with the purpose of this study, respondents were asked to provide an open-ended response to the following question: “Please describe any matters that you think need improvement in public health center operations for future pandemics.”

### Data analysis

We employed a text mining method to analyze the descriptions in three steps [20].

First, we performed a morphological analysis using ChaSen, a Japanese dictionary for text mining, to observe the 200 most frequently used words. To gain a comprehensive understanding of the descriptions, we also read all responses containing each word.

Second, we analyzed the descriptions using the topic model. A Topic model in text mining refers to the process of identifying key themes within a large body of text. This is typically achieved using statistical methods to analyze words and their frequency in a document, generating topics that signify the main themes present in the text. The number of topics was determined using perplexity, a measure of the model’s predictive performance [21]. In general, the lower the perplexity score, the better the model is considered. In this study, we used Latent Dirichlet Allocation for semantic interpretation, thereby identifying meaningful topics within our text corpus [22]. Through this approach, we were able to reveal hidden thematic structures, aiding in a more profound understanding of our data.

Third, based on the results of the topic model, we conceptualized and structured the issues to be discussed about the future health center. In the process of structuring, we observed the words in each topic and the actual descriptions in which they were frequently used.

For our analysis, we used the freely accessible text mining software, KH Coder 3.0, developed by Higuchi from Ritsumeikan University, Kyoto, Japan (https://khcoder.net/) [23]. The choice of this software was influenced by numerous successful instances of its usage in public health-related text mining studies [24, 25]. Alongside this, we utilized the ChaSen Morphological Analyzer, a Japanese dictionary for text mining, and the R statistical software environment for our analysis.

### Ethics

The study was structured to ensure that respondents reviewed and understood the guidelines about the study’s aim, significance, methodology, measures for personal information protection, and potential risks before proceeding to the questionnaire. The questionnaire was designed to be anonymous, reducing the risk of data leaks, and to enable respondents to freely express their honest opinions. Thus, the aim was to secure their understanding and consent before response, while ensuring the integrity of their responses and personal data.

This study adhered strictly to the principles of the World Medical Association Declaration of Helsinki and received approval from the University of Occupational and Environmental Health, Japan’s Ethics Committee (R4-042).

## Results

### Characteristics of respondents

The characteristics of the respondents are presented in Table 1. Of the 784 respondents, 494 (63.0%) were women. Respondents in their 40 s and 50 s comprised over 50% of the respondents. More than 50% of the respondents had worked continuously for more than 16 years. Conversely, 181 (23.1%) had been working for less than 5 years. Public health nurses and midwives accounted for 330 (42.1%), and managers for 285 (36.4%). Workplaces were public health centers for 662 (84.4%) and local governments for 114 (14.5%). Jurisdictions were spread across the country.

**Table 1:** Characteristics of respondents

	N	%
Sex		
Women	494	63.0
Men	290	37.0
Age, years		
20–29	131	16.7
30–39	141	18.0
40–49	162	20.7
50–59	274	34.9
60-	76	9.7
Length of continuous employment, years		
0–5	181	23.1
6–10	98	12.5
11–15	85	10.8
16-	420	53.6
Occupation		
Doctor, dentist	111	14.2
Public health nurse, midwife	330	42.1
Administrative staff	172	21.9
Other technicians	171	21.8
Job position		
Manager	285	36.4
Non-manager	499	63.6
Workplace		
Public health center	662	84.4
Local government	114	14.5
Other	8	1.0
Area of jurisdiction		
Hokkaido, Tohoku	144	18.4
Kanto	157	20.0
Chubu	135	17.2
Kinki	143	18.2
Chubu, Shikoku	76	9.7
Kyushu, Okinawa	129	16.5

### Morphological analysis

In the first step, a morphological analysis of the descriptions was conducted. In total, 36,632 extracted words and 3,075 unique words were identified. We set public health center(*hokenjo*), public health nurse(*hokenshi*), occupational physician(*sangyoi*), COVID-19(*korona*), and medical institution(*iryou-kikan*) as specific words that frequently occurred in this study and were not included in Chasen. The 50 most frequent words and their occurrence counts are shown in Table 2. These included operation, manpower, coordination, outsourcing, hospitalization, information, regular duties, survey, and policy.

**Table 2:** Top 50 frequent words and their number of occurrences

No	Word	Counts
1	Operation	421
2	Public health center	397
3	Response	357
4	Staff	261
5	Need	260
6	COVID-19	236
7	Think	175
8	Infectious disease	164
9	System	150
10	Manpower	129
11	Feel	115
12	Many	96
13	Burden	87
14	Conduct	85
15	Consider	84
16	Professional	79
17	Coordination	79
18	Deployment	79
19	Nation	77
20	Securing	75
21	Public health nurse	73
22	Medical institution	71
23	Assistance	67
24	Outsourcing	66
25	Management	66
26	Hospitalization	66
27	Improvement	58
28	Patient	57
29	Work	57
30	Time	57
31	Infection	56
32	Situation	56
33	Health	55
34	Administration	54
35	Medical Care	53
36	Countermeasure	53
37	Information	52
38	Responsibilities	48
39	Person	45
40	Regular duties	45
41	Crisis	44
42	Phone	44
43	Outbreak	44
44	Human resources	42
45	Organization	41
46	Large	41
47	Local government	40
48	Role	40
49	Survey	39
50	Policy	39

### Topic model analysis

**Fig. 1:**
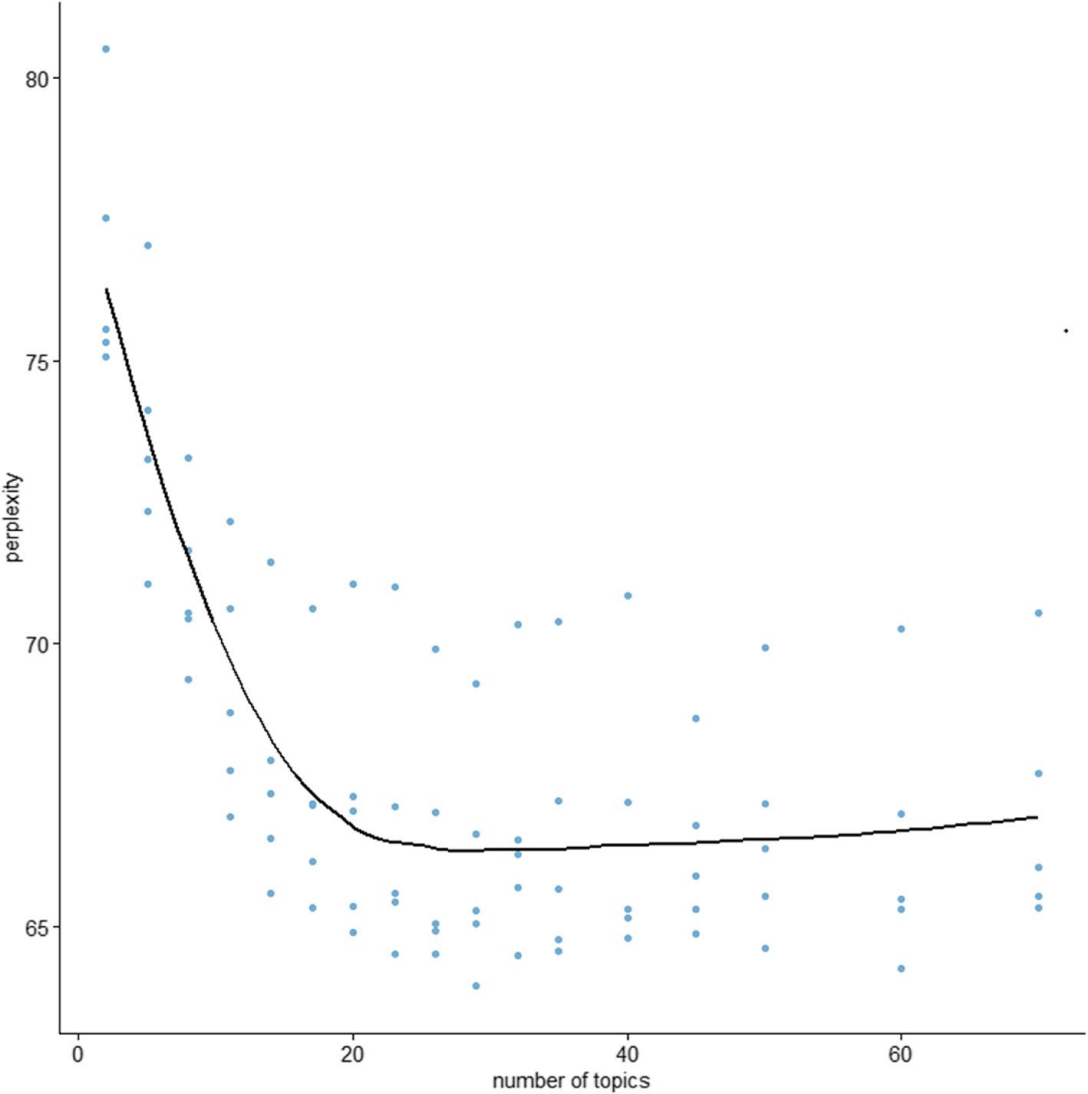
Relationship between the number of topics and perplexity

The results of the simulations for identifying the number of topics are shown in Fig. 1. We established the number of topics as 20. Table 3 displays the word sets that depict each topic when 20 topics were assigned. We interpreted the descriptions in which the word sets classified in each topic were frequently used and identified the issues raised by these descriptions.

**Table 3:** Topic numbers and words extracted from open-ended questionnaire responses

Number	Words
1	Infectious disease, Response, Department, Regular duties, Securing, Increase staffing, New type, Work, Responsibilities, Time
2	Response, Nation, Policy, Human resources, COVID-19, Community, On-site, Outsourcing, Role, Improvement
3	Public health nurse, Professional, Administration, System, Clerical position, Consider, Regular duties, Need, COVID-19, Securing
4	Response, Phone, Home care, Time, Facility, Duty, Case, Feel, Health, Understanding
5	Management, Crisis, Organization, Think, System, Case, Outsourcing, Large, Awareness, Person
6	Staff, Burden, Assistance, Large, Reduction, Increase staffing, Clerical position, Deployment, Person, Department
7	Operation, System, Think, Response, Infectious disease, Outsourcing, Understanding, Department, Manpower, Information
8	Public health center, Support, Person, Time, Awareness, Feel, Role, On-site, Securing, Large
9	Staff, Deployment, Health, Infectious disease, Nation, Person, Situation, System, Work, Responsibilities
10	Public health center, External, Review, On-site, Securing, Work, Infectious disease, Efficiency, Consider, Operation
11	Operation, Need, COVID-19, Staff, Regular duties, Outsourcing, Infection, Conduct, Administration, Responsibilities
12	Operation, Conduct, Efficiency, Survey, Situation, Outbreak, Health, Facility, Management, Many
13	Many, Countermeasure, Infection, Feel, Doctor, Situation, Administration, Understanding, Information, Work
14	COVID-19, Need, Think, Consider, Outsourcing, Conduct, Public health center, Survey, Professional, Medical institution
15	Response, Think, Feel, Assistance, Medical institution, Community, Staff, Outbreak, Consider, COVID-19
16	COVID-19, Feel, Information, Responsibilities, Especially, Assistance, Infection, Think, Role, Case
17	Coordination, Hospitalization, Medical Care, Patient, Medical institution, Consider, Public health center, Human resources, Improvement, Increase staffing
18	Manpower, Deployment, Improvement, Need, Feel, Awareness, Especially, On-site, Infectious disease, Review
19	Need, Infectious disease, Municipality, Work, New type, Securing, Medical institution, Information, Outbreak, Many
20	Operation, Public health center, Local government, Collaboration, Many, Improvement, Role, Need, Securing, Outbreak

### Conceptualization of discussion issues

We could summarize the descriptions into the following eight issues; 1) establishment of a crisis management system, 2) division of functions among public health centers, prefectures, and medical institutions, 3) clear role distribution in public health center staff, 4) training of specialists, 5) information sharing system (information about infectious diseases and government policies), 6) response to excessive workload (support from other local governments, cooperation within public health centers, and outsourcing), 7) streamlining operations, and 8) balance with regular duties. Table 4 lists these eight issues, the word sets of each topic that suggest these issues, and some representative descriptions from the respondents.

**Table 4:** Discussion points for considering improvements to the infectious disease pandemic with a focus on public health centers

Number	Theme	Topic^a^	Related words	Descriptions of questionnaire respondents
i	Establishment of a crisis management system	1	Infectious disease, Response, Department	*-We need a department specifically for COVID-19 response*
		5	Management, Crisis, System	*-Preparation for ‘health crisis management’ should be standard procedure, not a scramble when a crisis occurs. Prior to the pandemic, chronic understaffing and regular overtime were already issues, limiting the capacity to handle a health crisis on top of regular duties*
				*-In times of crisis, most leaders remain confined to traditional mindsets about departmental structures, inhibiting flexible response*
		9	Staff, Deployment, Nation, System	*-In our city, pharmacists and veterinarians are left to handle infectious diseases, with no public health nurses involved. Given that infectious disease control fundamentally involves public health, it’s vital to have public health nurses comprise 80% of staff handling infectious disease operations*
				*-The current staff is stretched thin, expected to cover a wide array of tasks. Given the protracted nature of this national emergency, it’s crucial to either develop the profrssional skill or establish a dedicated temporary department to deal with the pandemic, separate from regular public health center operations.”*
ii	Division of functions among public health centers, local governments, and medical institutions	10	Public health center, External, Review	*-We need to review the role of health centers in monitoring patients under home care*
				*-As of April 2023, a sudden change was made in the revised medical fee payment scheme, which required hospials to coordination with public health centers. This put a strain on public health centers, as they were expected to participate in conferences and take on administrative roles for the external medical association*
		17	Coordination, Hospitalization, Medical Care, Patient	*-When a doctor diagnoses a patient as requiring hospitalization, it should be the doctor’s role to cordinate a medical facility*
		19	Municipality, Medical institution	*-Public Health Centers were tasked with monitoring the health status of patients who, unable to be hospitalized, had to recuperate at home. We cannot provide medical examination or prescriptions even when they detect health issues. Thus, health observation should not be conducted by medical institutions*
		20	Operation, Public health center, Local government, Collaboration, Role	*-Understanding of each other’s tasks between the local government and public health center is crucial. There were frequent instances of frustration and unnecessary workload due to misunderstanding. Exchanging staff between two organizations for a certain period could improve the understanding of each other’s tasks and smooth the workflow*
				*-The division of authority between the local government and public health center is vague, leading to different directives coming from the leaders of each organization*
iii	Clear role distribution in public health center staff	3	Public health nurse, Professional, Administration, Clerical position	*-Professional staff are often overburdened due to the assumption that “specialized knowledge is needed,” even for tasks that are primarily administrative*
				*-During the COVID-19 crisis, it was unfeasible to distribute document-related tasks to a public health nurse. We believe that there is a necessity for dedicated administrative personnel*
		15	Response, Community, Staff,	*-The constant changes in COVID-19 responses made it difficult for those not directly involved in the pandemic efforts to grasp the entire picture. This created a constant anxiety about conveying incorrect information when fielding questions from hotline or citizens during*
				*-We have noticed a difference in thinking between the staff who are dedicated to handling COVID-19 matters and those who deal with it on a rotating basis*
iv	Training of specialists	7	Department, Manpower	*-We, the supporters, are also human beings. If initiatives that consider our own mental health are implemented, I believe many staff members can engage in their work more effectively*
				*-Inter-agency personnel exchanges could be considered to strengthen collaboration among different public health centers*
				*-In terms of the position of health center director, it shouldn’t merely be a doctor by default. Regardless of professional background, reforms are needed in the system to carefully select and assign individuals who are truly suitable for the role of director*
		19	Securing	*-Regarding the securing of public health doctors and planned staffing of public health nurses, it is crucial that local government leaders have a clear intention and move forward in securing these positions*
v	Information sharing system (information about infectious diseases and government policies)	2	Nation, Policy, Improvement	*-We were often thrown off course by the national government’s policy changes and slow decision-making. I wish they would refrain from issuing documents on Friday afternoons. Prefectures should formulate their own policies by considering both the national guidelines and local circumstances to protect their residents*
		13	Administration, Understanding, Information	*-Even though a lot of manuals and other materials are created by the national government, they often go unread. The sheer volume of information can make it difficult to understand what to pay attention to*
		16	Information, Responsibilities	*-There were frequent policy changes and a significant number of directives from the national and local governments. Even for us who only deal with COVID-19 related matters, it’s hard to keep up*
				*-Centralizing information dissemination to make the latest information easily understandable for everyone is necessary. Changes in national policy, variations in responses by different local governments, and each department having its own information for its respective duties caused confusion, especially during interactions with residents*
vi	Response to excessive workload (support from other local governments, cooperation within public health centers, and outsourcing)	4	Response, Phone	*-Receiving alerts from the fire department on my mobile phone during the night is both physically and mentally exhausting. Merely having my mobile phone with me all the time causes me to react to the ringtone on TV, and having the phone next to me while I sleep makes it impossible for me to mentally switch off*
				*-I have to respond to calls from emergency services, medical institutions, and patients, even in the middle of the night, while commuting, or on days off*
		6	Burden, Assistance, Increase staffing, Clerical position	*-Even if we increase the number of dispatched or assistant workers, if they don’t stay for long, we have to retrain each time. Ultimately, the primary staff have to make the final decisions and responses, which just increases their burden*
		8	Public health center, Support	*-The various policies that put pressure on public health centers in the name of “support” should be stopped*
		18	Manpower, On-site	*-In the midst of a staff shortage and swamped with processing immediate infectious patient reports, we did not have the capacity to consider improvements to our operations or secure additional manpower.I sincerely wished there were a team dedicated to handling on-site tasks and a team focused on improving operations*
				*-Given that our workplace consists predominantly of women, securing replacement personnel due to maternity and parental leave is challenging*
vii	Streamlining operations	12	Operation, Efficiency, Management	*-We should implement ICT across the entire agency and outsource tasks to streamline, consolidate, and improve work efficiency, while also distributing roles among related institutions*
				*-Even more than a year after the outbreak of COVID-19, public health centers were still conducting inefficient tasks such as phone interviews, handwritten memos, and data creation*
		15	Response, Staff	*-I was thrust into handling phone calls as staff without any prior job explanation, and in an environment where the phone never stopped ringing. With no manuals or guides, I was prompted to response on my own, which caused immense stress*
viii	Balance with regular duties	11	Regular duties	*-We frequently had to abruptly cancel regular duties due to changing circumstances. It was demotivating and a source of significant psychological stress to see the work we had built up suddenly revert back to square one*
		14	Outsourcing, Public health center	*-Juggling the responsibility of a COVID-19 duty role several times a month, in addition to regular duties, was extremely demanding emotional toll. Outsourcing to a dedicated agency would be beneficial*
				*-When tasks related to COVID-19 are present, it becomes impossible to perform the original duties of the public health center. I believe it would be beneficial to manage these tasks through outsourcing*

^a^The figures in the topic refer to numbers in Table 3

## Discussion

In our study, we identified following eight issues for the future pandemics: establishment of a crisis management system; division of functions among public health centers, local governments, and medical institutions; clear role distribution in public health center staff; training of specialists; information sharing system; response to excessive workload; streamlining operations; balance with regular duties. We were able to discover comprehensive issues that are not only internal to the public health center, but also external to related agencies.

In a crisis, it is first necessary to recognize that the situation is critical and to affirm that the situation differs from normal circumstances. This concept is addressed in the first issue: the establishment of a crisis management system. The same issues are raised for medical response to disasters, which are based on seven principles: command and control, safety, communication, assessment, triage, treatment, and transport, known by the initial abbreviation CSCATTT [26]. Among the eight key issues, both “Establishment of a crisis management system” and “Division of functions among public health centers, local governments, and medical institutions” pertain to command and control. Similarly, “Clear role distribution in public health center staff” is related to communication, while “Response to excessive workload” and “Balance with regular duties” are indicative of triage. Considering the similarity of the issues, leveraging existing disaster medicine frameworks and strategies could be useful in considering specific solutions for handling future pandemics.

The pandemic placed an overwhelming strain on public health centers [17]. Previous studies, like our own, have indicated that public health workers were forced to work long hours, with some required to be on-call at all hours, including nights and weekends. The lack of manpower prevented public health nurses from performing their regular duties such as supporting activities to prevent child abuse and neglect [27]. Despite attempts to increase the number of staff, securing full-time public health nurses proved challenging, resulting in an increase in part-time staff who couldn’t play a central role [28]. As highlighted in our study, the training and retention of specialists are crucial factors in pandemic response. Studies have shown that areas with a high number of public health nurses before the pandemic had fewer infections [29]. Securing human resources prior to an epidemic could be a crucial issue.

Beyond maintaining a system for normal operations, it is important to be prepared to organize people flexibly during a pandemic. It has been reported that during the pandemic, public health nurses in local governments helped to address the shortage of manpower in public health centers [30]. Given the financial constraints of maintaining sufficient human resources under normal circumstances, temporary support will be necessary. The establishment of a crisis management system, as raised in this study, has already begun in some places. In Japan, the Infectious Disease Health Emergency Assistance Team (IHEAT) system has been set up to provide external resources for public health centers. IHEAT is a system in which public health nurses and other professionals in the community support the work of public health centers during health crises, such as the spread of infectious diseases [31]. This project, which began during the pandemic, was formalized in the revision of the Community Health Law in April 2023. As a result, local governments that have established public health centers are now responsible for securing a support system with IHEAT personnel. For the future, we will need to prepare for a cooperative system that is tailored to the local circumstances.

Streamlining operations is also needed to reduce overwork during a pandemic. Numerous solutions that do not rely on human intervention, such as digital tools for epidemiological surveys and consultation services, have been developed during the pandemic [32–34].

COVID-19 led to a significant issue about infodemics due to the abundance of mixed information, which became a major public health issue [35]. In this study, some respondents stated difficulties in handling numerous phone calls from residents. Additionally, the changing nature of the infection situation compounded this challenge, as new government guidelines were frequently issued, leaving public health workers in a difficult situation with uncertainty about the latest information [6]. Implementing effective information sharing systems, especially about the government policy, would be useful in managing infodemics.

### Strengths and limitations

One significant strength of this study is that it was able to consolidate the issues that need to be addressed for the future health center system based on the opinions of a wide range of frontline workers. Our study included individuals working in local governments and public health centers across the country, which provided us with a variety of perspectives. Past studies have had limited generalizability, as most were conducted in a few public health centers and mainly involved public health nurses [9, 17]. Furthermore, the application of a topic model in our analysis added a level of objectivity to the interpretation by researchers.

However, this study also has limitations. The first limitation is in its representativeness. When we recruited respondents for the survey, we asked all public health centers and local government managers to cooperate with us to collect as many respondents as possible. The managers who agreed to cooperate then encouraged cooperative people in their facilities to respond. Therefore, it is possible that there was sampling bias, leaning toward groups who were supportive of the study or who had many requests for improvements in their management. We note that the characteristics of the respondents were not representative of the workers who responded to the pandemic. The second limitation involves verification of the reported information in the open-ended questions. We designed the anonymous questionnaire so that respondents could freely express their opinions. As a result, we were not able to verify the accuracy of the descriptions provided. Although this study successfully identified key issues in preparing for future pandemics, it underscores the need for more objective future surveys. Such a survey would help in effectively determining the prioritization and resolution of these identified issues. The third is the inherent subjectivity involved in the researchers’ interpretation of the data. Given the necessity of interpreting the responses when structuring them, we couldn’t achieve wholly objective structuring. However, this study mitigated this effect by using a topic model to identify notable responses and cross-verifying actual responses with co-authors.

## Conclusions

In contemplating the future of the public health center system, our findings suggest eight pivotal issues that warrant further discussion. Leveraging the experience accumulated during the pandemic to fortify the public health system will be essential moving forward.

### Supplementary Information

Supplementary Material 1.

## Data Availability

The datasets used and analyzed during the current study are available from the corresponding author on reasonable request.

## Abbreviations

COVID-19: The novel coronavirus disease 2019
IHEAT: Infectious Disease Health Emergency Assistance Team
